# A Neglected Case of Multifocal Liposarcoma Presented in Five Different Sites in a Syrian Woman: A case report

**DOI:** 10.1016/j.ijscr.2019.10.077

**Published:** 2019-11-05

**Authors:** Ieman Alawad, Ahmad Al-Haj, Ahmad Ghazal, Amna Ibrahim, Ruqaya Masri

**Affiliations:** a Aleppo University Hospital for Obstetrics and Gynecology, University of Aleppo, Aleppo, Syria; b Department of Surgery, University of Aleppo, Aleppo, Syria; c Department of Internal Medicine, University of Aleppo, Aleppo, Syria

**Keywords:** Liposarcoma, Round cell, Multifocal, Case report

## Abstract

**Introduction:**

Multifocal soft tissue sarcoma is a rare clinical entity occurring in 1 % of patients with extremity soft tissue sarcoma and in 4.5 % of patients with liposarcoma. Multifocal disease may arise either synchronously or metachronously and has been associated with poor prognosis.

**Case presentation:**

Herein, we report a rare case of a Syrian woman patient with six different foci of liposarcoma in five sites at the time of diagnosis.

**Discussion:**

Liposarcomas are currently classified into four different subtypes based on histologic or genetic analysis according to the World Health Organization (WHO), including cell-differentiated, dedifferentiated, myxoid, and pleomorphic. In the present work, we report a neglected patient with a multicentric round cell liposarcoma in five different sites.

**Conclusion:**

Medicine cannot provide much care for these advanced cases of multicentric liposarcoma so far. Therefore, more research should be conducted to improve the ability to manage these entities and to identify potential novel therapies.

## Highlights

• Complete surgical resection of liposarcoma is central in treatment based on grade.
• Radiation reduces risk of local recurrence or minimize surgical morbidity in the highly radiosensitive myxoid/round cell liposarcoma group.
• Chemotherapy is utilized in chemosensitive histologies with metastatic potential.
• Understanding of the genetic aberrations allows development of targeted therapies for liposarcoma.

## 1 Introduction

Liposarcoma is a common malignant soft tissue tumor, accounting for 10 %–16 % of all sarcomas. It typically affects patients between the fifth and seventh decade of life and usually develops in the extremities or retroperitoneum [1].

Multifocal disease is a rare clinical entity occurring in 1 % of the patients with extremity soft tissue sarcoma [2,3] and in 4.5 % of the patients with liposarcoma [3,4].

Multifocality in soft tissue sarcoma is defined as the development of a soft tissue sarcoma in two or more separate anatomical sites before the manifestation of the disease in common metastatic sarcoma sites, particularly in the lungs [3,4].

There are no significant differences in sex predilection, age, grade, and depth margins between multifocal and unifocal disease [2,3].

They can be either synchronous or metachronous and are generally associated with an aggressive clinical course and poor prognosis [3,5].

These fat tumors, of ubiquitous localization, commonly appear as a slowly enlarging mass with a misleadingly benign appearance. However, any soft-tissue tumor requires the need for a thorough preoperative X-Ray investigation [6] and a biopsy should be performed if of more than five centimeters in diameter [7].

Herein, we report a rare case of a Syrian female patient with six different foci of liposarcoma in five locations that developed six months after the resection of a thigh liposarcoma.

This work has been reported in line with the SCARE criteria [8].

## 2 Case presentation

A 37-year-old Syrian woman came to our clinic complaining of four large masses in the leg, thigh, neck, and abdomen. The complaint started 30 months ago as a small hard mass in the upper of the right thigh. The patient underwent a complete resection of the mass. Histological examination showed that it was a liposarcoma. Six months later, other four masses appeared in abdomen, neck, thigh, and leg. The masses showed rapid growth. During the last twenty-four months, the patient was neglected because of the crisis that affected every part of Syria. She was not followed up by regular imaging. Even after the masses were visible and large, she did not receive any therapy until she got presented to our clinic. Her family history was remarkable. Her brother and sister had lipomas. However, genetic studies were not performed because they are expensive and not available in our country. Furthermore, her drug and psychosocial history was passive.

**Fig. 1:**
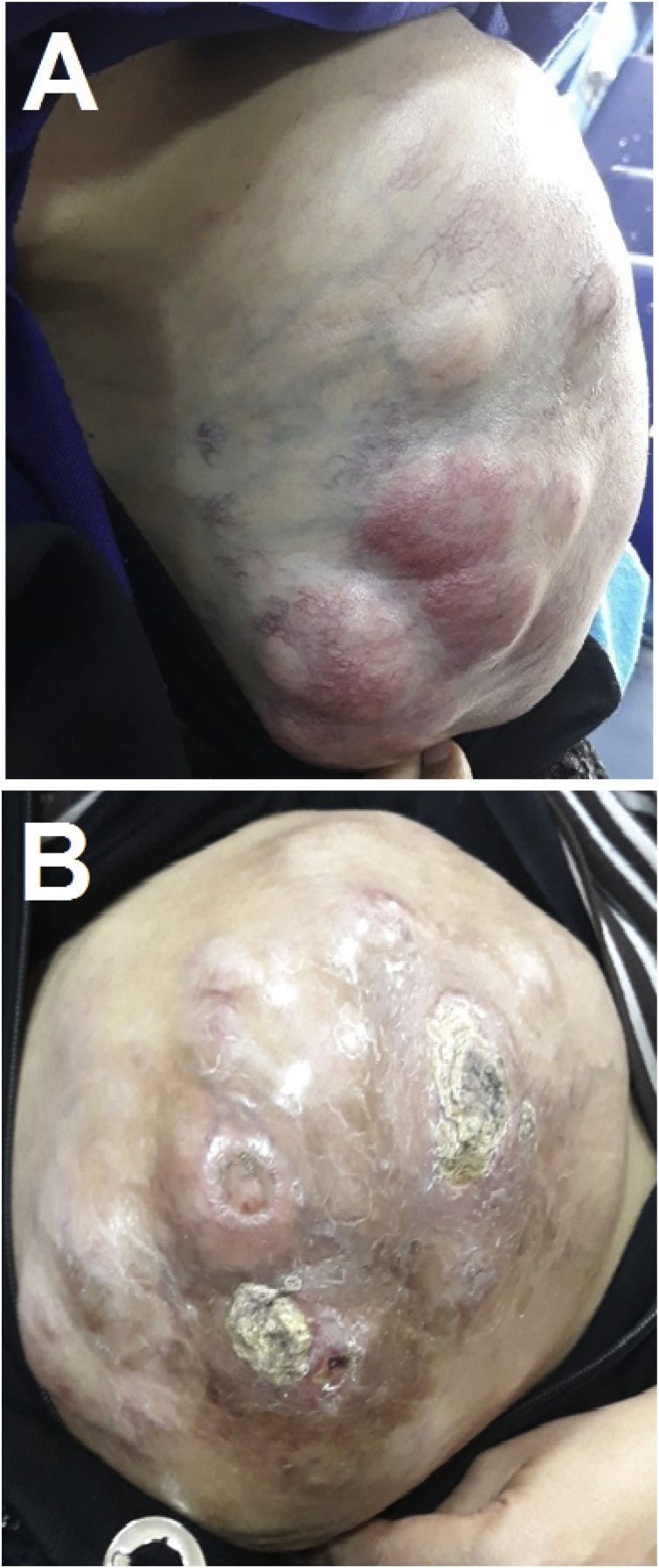
Two large multilobulated masses: A. Left laterocervical mass. B. An ulcerated subcutaneous mass in the abdomen.

**Fig. 2:**
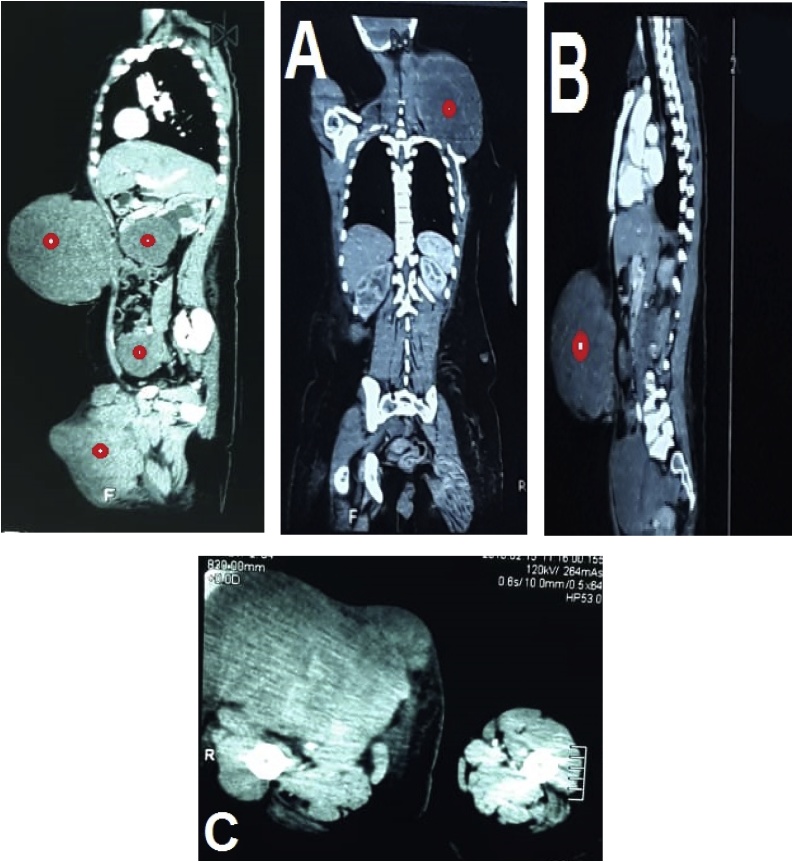
CT scan demonstrating four homogenous masses with low density consisting with liposarcoma. A. The left laterocervical mass measuring 12 × 22 × 18 cm. B. The subcutaneous and retroperitoneal masses that measure 23 × 19 × 16 cm, 8 × 8 × 8 cm respectively. C. The upper outer right thigh measuring 20 × 21 × 25 cm.

Clinical examination revealed that the masses were hard, fixed and large (Fig. 1). Full blood count and biochemical investigations were within the normal range. Computed tomography (CT) showed masses in the abdomen, a large subcutaneous mass and a retroperitoneal mass, the pelvis, the neck and the thigh. The left cervical mass was invading the root of the neck, the cervical plexus, and the subclavicular artery. The upper outer right thigh mass was invading the femoral artery. All the masses had a low density, which was consistent with a lipomatous tumor (Fig. 2).

**Fig. 3:**
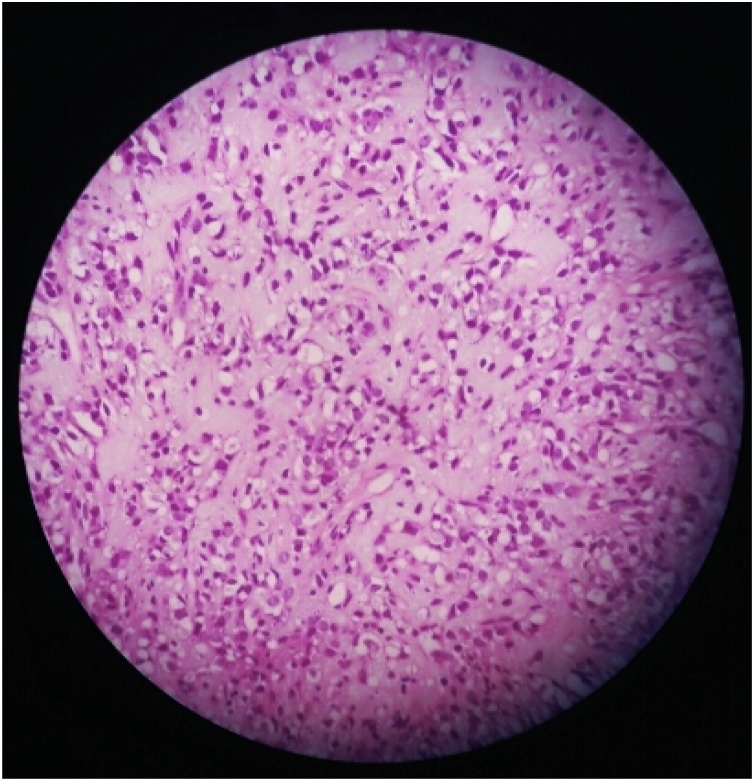
High-grade liposarcoma with prominent round cell component (H&E × 400).

A surgical biopsy from the thigh mass showed high-grade liposarcoma with prominent round cell component (Fig. 3).

**Fig. 4:**
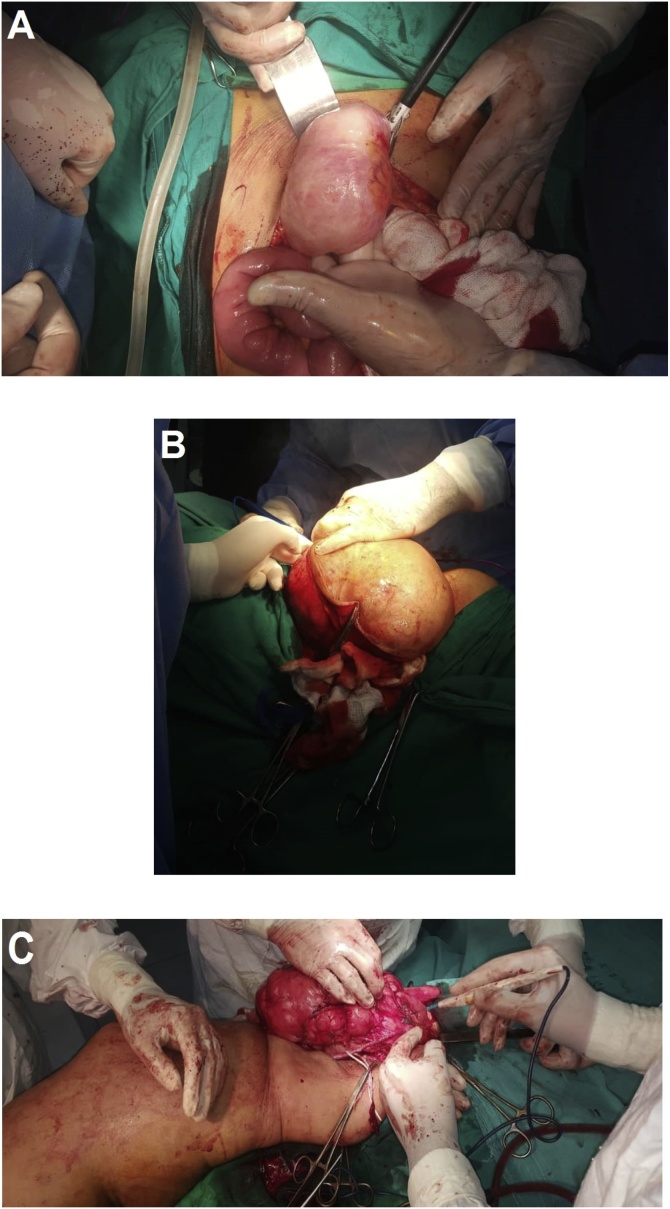
A. Abdominal masses resecting. B. Cervical mass resecting. C. femoral mass resecting.

Because of rapid recurrence and multifocal disease, the multidisciplinary team suggested chemotherapy with Doxorubicin and Ifosfamide. The patient was given five cycles of chemotherapy. Postchemotherapy follow-up showed a decreasing by 30 % in the masses size. Two months after chemotherapy, the patient underwent two major surgical operations. The first one was for resecting abdominal and cervical masses and the other one was for resecting femoral and popliteal masses (Fig. 4). Actually, the patient showed patience and she accepted all the offered treatments and procedures with satisfaction.

The first procedure was performed by a general surgery specialist with 15 years of experience in abdominal surgery. Abdominal incision showed seven tumor masses; a large retroperitoneal mass, an under left kidney mass, a peripheral retroperitoneal mass connected to the upper pole of the right kidney and compressing the inferior vena cava, two pelvic masses adherent to the right ovary and greater omentum, a small mass connected to the transverse colon, and a mass invading the sigmoid colon mesentery. They measured 30, 5, 10, 15, 5, 4 and 10 cm respectively. Therefore, the resectable masses were totally resected and the other masses were debulked. The cervical mass was only debulked to save neural and vascular elements and to avoid extremity amputation.

Seventeen days later, the femoral and popliteal masses were resected after isolating the neurovascular bundles and nerves. This procedure was performed by an orthopedic surgery specialist with 10 years of experience in extremity cancer surgery.

The pathologic report was liposarcomatosis (myxoid type). Unfortunately, a day after surgery the vital signs of the patient deteriorated. She was transformed to the surgical intensive care unit (ICU) where she died.

## 3 Discussion

Liposarcomas are currently classified into four different subtypes based on histologic or genetic analysis according to WHO, including cell-differentiated, dedifferentiated, myxoid, and pleomorphic [9]. The round cell subtype is now representative of a higher grade of the myxoid variant. The myxoid variant accounts for one-third to one-half of all liposarcomas, commonly occurring in a younger demographic with a peak incidence in the fifth decade, in comparison to the dedifferentiated and well-differentiated subtypes which demonstrate peak incidence in the seventh decade. The most common sites of tumor involvement include the lower extremities with a predilection for the medial thigh and popliteal area [10].

As myxoid and round cell tumors share the same cytogenetic abnormalities, namely the translocation t (12; 16) (q13; p11) leading to the fusion of the genes DDIT3 and FUS with the generation of a hybrid protein FUS/DDIT3, some authors consider both lesions as a continuum of the same disease. This possibility seems to be supported by the frequent finding of areas of round cells in myxoid liposarcomas, which has been considered a marker of poor prognosis when representing 5 % or more of the mass in localized myxoid liposarcoma [5].

With our patient, the specimen was not suited for cytogenetic analysis.

This entity seems to be rather infrequent and some authors consider it different from ordinary liposarcoma due to both multicentric presentation and more aggressive behavior [5].

The first reported case of multifocal soft tissue sarcoma dates back to 1934 when Siegmund described a patient with multiple fatty tumors and coined the term “Lipoblastische Sarcomatose.” Since then, less than 50 cases have been reported in the literature, mostly in the form of case studies. The largest series was reported in 1962 by Enzinger, who described 20 cases in whom the tumors were exclusively of the myxoid and round cell types, exhibiting a different pattern of spread [3].

Debate still exists as to whether this entity represents a rare variant of an already rare disease or whether it is simply a more unusual metastatic pattern [2].

Interestingly the multicentric tumors tend to spare classical metastatic sites of sarcomas, like the liver, the lungs or the bone and affect rare locations, like the pleura or the lymph nodes, but in our patient; the previous sites were not affected. This fact seems against a possible metastatic explanation for multicentricity. The identification of the same cytogenetic abnormalities in all the multicentric lesions cannot be considered either definite proof of their metastatic origin, for they might still be multicentric synchronous or metachronous lesions related to a common aetiopathogenic factor [5].

In all three groups, complete surgical resection is central in treatment aimed at cure and is based on grade. Radiation can reduce risk of local recurrence in high-grade lesions or minimize surgical morbidity in the highly radiosensitive myxoid/round cell liposarcoma (M/RCLS) group. The biologic groups differ greatly in their chemosensitivity, so adjuvant chemotherapy is selectively utilized in chemosensitive histologies with metastatic potential (i.e. round cell and pleomorphic liposarcomas) but not in the relatively resistant subtype dedifferentiated liposarcoma (DDLS). An improved understanding of the genetic aberrations that lead to liposarcoma initiation is also allowing for the rapid development of targeted therapies for liposarcoma. Among such therapies are CDK4 inhibitors in well- and dedifferentiated liposarcoma (WD/DDLS) and trabectedin, which prevents FUS-DDIT3 binding to DNA, in (M/RCLS) [9].

With our patient surgery was not performed initially because of rapid recurrence and infiltration of large vessels and neural plexus, so that she was put on a palliative chemotherapy. After chemotherapy, surgical treatment was done.

As prognosis seems to be poor, chemotherapy and radiotherapy seem indicated both in the neoadjuvant or adjuvant settings. As these multicentric tumors are rare, it is difficult to determine which regimen could be the best in these patients. Nevertheless, the literature does not indicate a significant improvement of the outcome regardless of the chosen therapy [5].

## 4 Conclusion

In the present work, we report a neglected case of multicentric liposarcoma that developed in five different sites after the resection of the primary mass. For these advanced cases, medicine cannot provide much care so far and prognosis remains poor. So, we should conduct more research to improve our understanding of how each subtype of tumor responds to different therapies. By that, we can manage these entities and identify potential novel therapies.

Family members should undergo periodic genetic tests for early detection of the potential cases, so they can receive early treatment.

We should also draw attention to the importance of establishing referral centres to provide treatment to such advanced cases.

These cases should be managed by a multidisciplinary team in order to increase the opportunity of total recovery and to reduce recurrence.

Unfortunately, such advanced cases are a result of decreased general healthcare which is expected in time of crisis as happened in our country.

## Funding

The authors declare that the research have no funding sources or sponsors.

The authors requested a full waiver for the article processing charges.

## Ethical approval

Not required for case reports at our hospital. Single case reports are exempt from ethical approval in our institution.

## Consent

Written informed consent was obtained from the patient for publication of this case report and accompanying images. A copy of the written consent is available for review by the Editor-in-Chief of this journal on request.

## Author’s contribution

**Ieman Alawad, Amna Ibrahim, Ruqaya Masri**: Design the study, data collection, drafting, revision.

**Ahmad Al-Haj, Ahmad Ghazal**: The Supervisors, patient care, revising critically.

All authors discussed the content of the manuscript, read and approved the final manuscript.

## Registration of research studies

Not applicable. This case is not a part of any clinical study.

## Guarantor

Dr. Ieman Alawad.

## Provenance and peer review

Not commissioned, externally peer-reviewed.

## Declaration of Competing Interest

The authors declare that they have no conflicts of interest.
